# Endoscopic management of a sessile serrated lesion at the appendiceal orifice

**DOI:** 10.1055/a-2699-9086

**Published:** 2025-10-09

**Authors:** Jingjing Yao, Feifei Zhang, Wen Jiao, Hongyuan Cui, Jindong Fu

**Affiliations:** 1 549615 Department of Gastroenterology, Rizhao People's Hospital, Rizhao, China; 2 576398 Affiliated Hospital of Shandong Second Medical University, Weifang, China; 3 654581 Jining Medical University Clinical Medical College, Jining, China

**Fig. 1:**
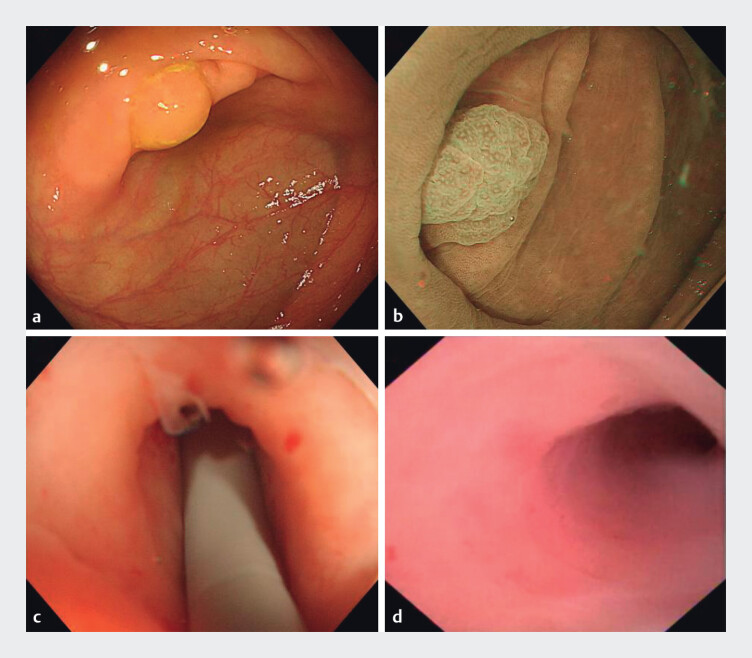
Endoscopic images of a lesion and the appendix lumen. **a** A flat lesion at the appendiceal orifice, capped with yellow mucus. **b** Type IIo glandular structures under narrow-band imaging (NBI). **c,d** The mucosa within the appendix was smooth.

A 36-year-old woman with no history of appendectomy was found to have a 1.5-cm flat lesion at the appendiceal orifice during routine colonoscopy. The lesion, capped with yellow mucus ( Fig. 1 **a** ), had type IIo glandular structures under narrow-band imaging (NBI) ( Fig. 1 **b** ). Due to the coverage by the Gerlach valve, it was unclear whether the lesion extended into the appendix lumen.

To further evaluate the lesion, a cholangioscope was introduced into the appendix lumen. The mucosa within the appendix was smooth and the lesion was found to extend only to the appendiceal orifice without involvement of the lumen ( Fig. 1 **b** , Fig. 1 **c** ). After obtaining informed consent, endoscopic submucosal dissection (ESD) was performed for resection ( Video 1 ).

Endoscopic management of a sessile serrated lesion at the appendiceal orifice.Video 1

**Fig. 2:**
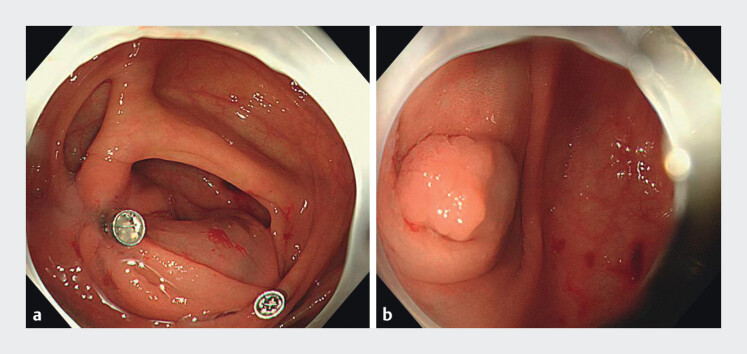
Pulling and fixing of the valve to the colonic wall to expose the margin of lesions. **a** Two harmonic clips were used to pull and fix the valve to the colonic wall. **b** Lesion margins were completely exposed.

**Fig. 3:**
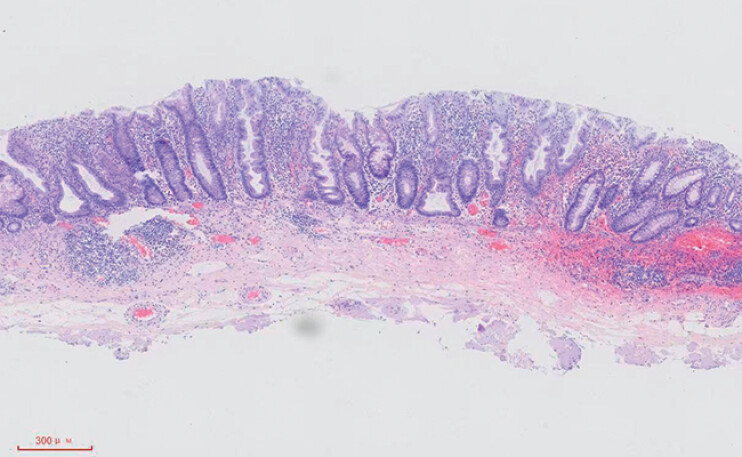
Postoperative histopathological analysis confirmed that the lesion was sessile and serrated.

To minimize coverage by the Gerlach valve, two harmonic clips were used to pull and fix the valve to the colonic wall, directing it toward the anal side ( Fig. 2 **a** ). This method facilitated exposure of the lesion margins ( Fig. 2 **b** ). After circumferential dissection, the lesion was completely excised using a traction method. After thorough hemostasis, the wound was left unsealed. Postoperative histopathological analysis confirmed presence of a sessile serrated lesion (SSL) with negative surgical margins ( Fig. 3 ). The patient was placed on a 48-hour fast following the procedure and was discharged in good condition 3 days later.

Sessile serrated lesions (SSLs) are predominantly located on the right side of the colon 1 and may extend to the appendix 2 . Our case demonstrates that when an SSL is located at the appendiceal orifice and it is unclear whether the lesion extends into the appendix lumen, a cholangioscope can be employed for assessment to determine the subsequent treatment approach, including whether an appendectomy is needed. In addition, our experience shows that using harmonic clips to pull the Gerlach valve can help better expose the lesion in the ileocecal recess, facilitating treatment.
